# A systematic review assessing non-pharmacological conservative treatment studies for people with non-inflammatory multi-joint pain: clinical outcomes and research design considerations

**DOI:** 10.1007/s00296-017-3876-1

**Published:** 2017-11-16

**Authors:** C. Comer, T. O. Smith, B. Drew, R. Raja, S. R. Kingsbury, Philip G. Conaghan

**Affiliations:** 1 Extended Scope Physiotherapy Practitioner, Leeds Community Healthcare Musculoskeletal and Rehabilitation Services, Leeds, UK; 2 Physiotherapy, School of Health Sciences, University of East Anglia, Norwich, UK; 3 Leeds Institute of Rheumatic and Musculoskeletal Medicine, University of Leeds, Leeds, UK; 4 Christchurch Hospital, Christchurch, New Zealand; 5 NIHR Leeds Biomedical Research Centre, University of Leeds, Leeds, UK

**Keywords:** Pain, Arthritis, Therapeutics, Clinical Trial, Research design

## Abstract

To systematically review the evidence to determine the clinical outcomes and the important methodological quality features of interventional studies on adults with non-inflammatory multi-joint pain (MJP). Systematic search of published and unpublished literature using the databases: AMED, CINAHL, MEDLINE, EMBASE, psycINFO, SPORTDiscus, PEDro, OpenGrey, the EU Clinical Trials Register, World Health Organization International Clinical Trial Registry Platform, ClinicalTrials.gov and the ISRCTN registry (search: inception to 19th October 2017). All papers reporting the clinical outcomes of non-pharmacological interventions for people with non-inflammatory MJP were included. Studies were critically appraised using the Downs and Black Critical Appraisal and the TIDieR reporting checklists. Data were analysed using a Best Evidence Synthesis approach. From 3824 citations, four papers satisfied the eligibility criteria. Three studies reported outcomes from multidisciplinary rehabilitation programmes and one study reported the findings of a spa therapy intervention. All interventions significantly improved pain, function and quality of life in the short-term. There was limited reporting of measures for absenteeism, presenteeism and psychosocial outcomes. The evidence was ‘weak’, and due to a lack of controlled trials, there is limited evidence to ascertain treatment effectiveness. Design consideration for future trials surround improved reporting of participant characteristics, interventions and the standardisation of core outcome measures. There is insufficient high-quality trial data to determine the effectiveness of treatments for non-inflammatory MJP. Given the significant health burden which this condition presents on both individuals and wider society, developing and testing interventions and accurately reporting these, should be a research priority.

*Registration* PROSPERO (CRD42013005888).

## Introduction

Musculoskeletal pain presents a significant clinical challenge and is associated with a substantial health and social burden [1, 2]. The majority of patients with musculoskeletal complaints experience pain at more than one joint [3, 4]. Non-inflammatory multi-joint pain (MJP) represents a complex mix of osteoarthritis, back pain and soft tissue disorders [5]. It is associated with increased disability, depression and lost work productivity [6]. People with MJP may have a median of six painful joints [5] and evidence indicates that an increasing number of painful joints is associated with poorer physical and mental statuses [7, 8], increasing the risk of restrictions on both activity and social participation. The costs associated with MJP are significantly greater than those associated with low back pain alone, which costs the NHS approximately £1700 million annually [2].

Both clinical care and research have traditionally focused on treating single joint pain and disability [9], failing to recognize the impact of MJP on treatment choices and outcomes [10]. Whilst a recent survey suggests that the majority of general practitioners now treat MJP concurrently rather than focusing on a single joint [11], there remains uncertainty on how this should be operationalized, and what interventions should be used. To this end, the National Institute for Health and Care Excellence (NICE) recommended in their osteoarthritis guidelines [12] that trials to investigate interventions for the management of MJP should be a research priority.

Currently, only one systematic review has examined interventional trials for people with MJP [9], concluding that there is limited evidence to guide treatment choice. However, whilst all studies in this review investigated the effectiveness of an intervention package designed for people with MJP, in practice, participants were recruited into these trials with single joint pain in the hip, knee or hand joints [13–15] rather than targeting people with co-existing pain in two or more joints. Furthermore, this review paper was based only on studies which were multidisciplinary in delivery (two or more different health professional groups), were delivered in primary or community care settings, and were required to incorporate NICE recommended core treatments [12]. Consequently, it remains unclear whether outcomes for these interventions would differ with a true MJP population, and if there are other interventions which may be supported for this population when delivered by specific professional groups in secondary and primary care.

The purpose of this review is, therefore, to: (1) address this uncertainty and examine the current literature to determine the effectiveness of different interventions for people with non-inflammatory MJP, and (2) identify key research design features which should be considered when designing future trials on people with non-inflammatory MJP.

## Methods

### Search strategy

The primary search was of published literature searching the databases: AMED, CINAHL, MEDLINE, EMBASE, psycINFO, SPORTDiscus and PEDro. Secondary search strategies included searching the unpublished and grey literature databases: OpenGrey, the EU Clinical Trials Register, World Health Organisation International Clinical Trial Registry Platform (ICTRP), ClinicalTrials.gov, and the ISRCTN registry. All databases were searched from database inception to 19th October 2017, and performed by one reviewer (TS). The search strategy for the MEDLINE search (via Ovid) is presented in Table 1. This was modified for each individual database. The reference lists from all potentially eligible papers and review papers were reviewed. All corresponding authors from each included study were contacted to review the search results to identify any additional studies which may have been initially omitted.

**Table 1:** MEDLINE search strategy

1. joint diseases/
2. arthropathy.ti,ab
3. arthritis/
4. esp osteoarthritis/
5. (pain$ adj3 (dual$ widespread or many)).ti,ab
6. (pain$ adj3 (number or one or two or three or four) adj3 (site$ or location$ or area$ or joint$)).ti,ab
7. (pain$ adj3 (multi$ or multi?joint or multi?site or multi?focal)).ti,ab
8. ((multi$ or widespread or dual$) adj5 (musculo?skelet$ or joint$) adj5 (pain$ or problem$)).ti,ab
9. generalized osteoarthritis.ti,ab
10. generalized pain.ti,ab
11. widespread pain.ti,ab
12. musculoskeletal pain.ti,ab
13. widespread musculoskeletal pain.ti,ab
14. multisite musculoskeletal pain.ti,ab
15. multiple pain sites.ti,ab
16. regional pain.ti,ab
17. fibromyalgia/
18. fibromyalgia.ti,ab
19. OR/2–18
20. AND/1,19

### Eligibility criteria

Studies were eligible if they satisfied the following criteria.

#### Design

Randomized or non-randomized trials presenting clinical outcomes for one or more defined interventions for the population of interest. Data on the location of intervention, who delivered it and the frequency to which it was provided was the minimum information required to be defined as an intervention. We excluded all basic science research and animal studies.

#### Population

Adults (16 years and over) with concurrent pain located at two or more joints e.g. ankle and hip. In accordance with Raja et al. [5] definition of MJP, we defined a joint-site as a region e.g. hand, foot, rather than by individual ‘small’ joints such as 1st carpometacarpal and 5th proximal interphalangeal joint. It could include combinations of joint disorders including osteoarthritis, back pain and tendinopathy. Pain could be a self-reported and/or physical examination-based diagnosis, but we excluded studies where MJP was diagnosed solely by radiological investigation. We excluded studies where participants reported pain in two or more locations without specific joint involvement such as fibromyalgia, myofascial pain, or widespread pain originating from soft-tissue/connective tissue disorders. “Methods” or “Results” section required to clearly indicate concurrent involvement of two or more joints. If the term “and/or” for joint involvement was used in the methodology, the “Results” section must have described concurrent involvement of two or more joints. e.g. ‘ankle and hip joint pain’ or ‘five percent of participants had pain in two joints’. However, we included studies which recruited people with MJP and non-MJP where the population with MJP was specifically identified and results for the cohort were reported separately, or where 90% or over of that cohort were diagnosed with MJP. We excluded all studies where participants had inflammatory arthropathies such as rheumatoid arthritis.

#### Intervention

Any non-pharmacological interventions or care pathways for the population of interest were included. This, therefore, included exercise and physical activity interventions, pacing and behavior modification interventions, psychological interventions, self-management programmes and device/assisted technologies. Packages of care which included one or more of these interventions were included. Interventions which comprised both a pharmacological and non-pharmacological treatment were included when the principle intervention was non-pharmacological and the pharmacological treatment an adjunct. We did not place a restriction on the frequency or intensity of an intervention, the location of delivery or who (which professionals) delivered the intervention.

#### Comparison

Any intervention or non-treatment control group was eligible as a comparator. Papers which did not include a comparator group (i.e. pre-post test design) were included.

#### Outcome

The a priori primary outcome measure was pain at 6-months post-commencement of the intervention. This could have been measured as part of a tool such as the Western Ontario and McMaster University Arthritis Index score (WOMAC) [16] or as a numerical rating scale (NRS) or visual analogue scale (VAS) pain score.

Secondary outcome measures were:

- Pain measured at other time-points,
- Physical function measured with tools such as the WOMAC [16], Knee Injury and Osteoarthritis Outcome Score (KOOS) [17] or Oswestry Disability Index scores (ODI) [18],
- Health-related quality of life measured with instruments such as the Short Form-12 (SF-12) [19], SF-36 [20], or the EQ-5D-5L [21],
- Anxiety and depression measured with tools such as the Hospital Anxiety and Depression scale (HADS) [22],
- Fear avoidance and kinesphophia measured with tools such as the Fear Avoidance Beliefs Questionnaire [23] and Tampa Scale of Kinesphobia [24],
- Self-efficacy measured with tools such as the General Self-Efficacy Scale [25],
- Work absenteeism and presenteeism.

Assessment intervals were defined as immediate-term (0–6 weeks), short-term (more than 6 weeks–3 months), mid-term (more than 3–12 months) and longer-term (more than 12 months).

#### Publication

We included all papers which reported eligible trials, irrespective of date of publication, language of publication, or where (geographically) the study was conducted.

### Study identification

Three reviewers (RR, BD, CC) independently reviewed the titles and abstracts of all search results using the defined eligibility criteria. Full-texts for all papers deemed potentially eligible were gathered, and re-reviewed by the three reviewers (RR, BD, CC) for full eligibility. All papers satisfying the criteria and agreed between two or more reviewers were included.

### Data extraction

Data extracted included: population characteristics definition of MJP; participant: age, gender, joint pain location, number of joint pain sites, BMI, SES group; country of trial origin; sample size; location of intervention delivery (i.e. primary or secondary care); intervention constituents i.e. treatment types, dose and frequency, self-management/home management programme, co-interventions; control intervention constituents; outcome measures and follow-up intervals assessed; length of follow-up; clinical findings including effect size and intervention fidelity. All data were collected independently onto a pre-defined data extraction table by three reviewers (BD, CC, TS). Any disagreements were resolved through discussion.

### Critical appraisal

To assess the quality of the current evidence and key design features for trials of people with MJP, all included studies were critically appraised using the Downs and Black Checklist [26]. This is a reliable and valid critical appraisal tool for non-randomised and randomised controlled trials [26]. It includes 27-items assessing: reporting, external validity, internal validity and power. To assess the reporting of study interventions we used the 12-item TIDieR checklist [27]. This assesses: intervention reporting by asking questions on: why, what (materials), what (procedure), who provided, how, where, when and how much, tailoring, modifications, how well (planned), how well (actual) [27]. The assessment for both checklists was independently performed by two reviewers (BD, CC).

### Data synthesis

Study heterogeneity was assessed by examining the data extraction table. Due to between-trial variability in cohort participant’s characteristics, interventions (experimental and control) and study design/processes, a meta-analysis was inappropriate [28]. Consequently, a narrative analysis of the data was performed using a Best Evidence Synthesis approach [29] where studies were graded as ‘strong’, ‘moderate’ or ‘weak’ as a judgement made by the three reviewers (CC, BD, TS) based on the Downs and Black assessment and the TIDieR evaluation.

## Results

### Search strategy

**Fig. 1:**
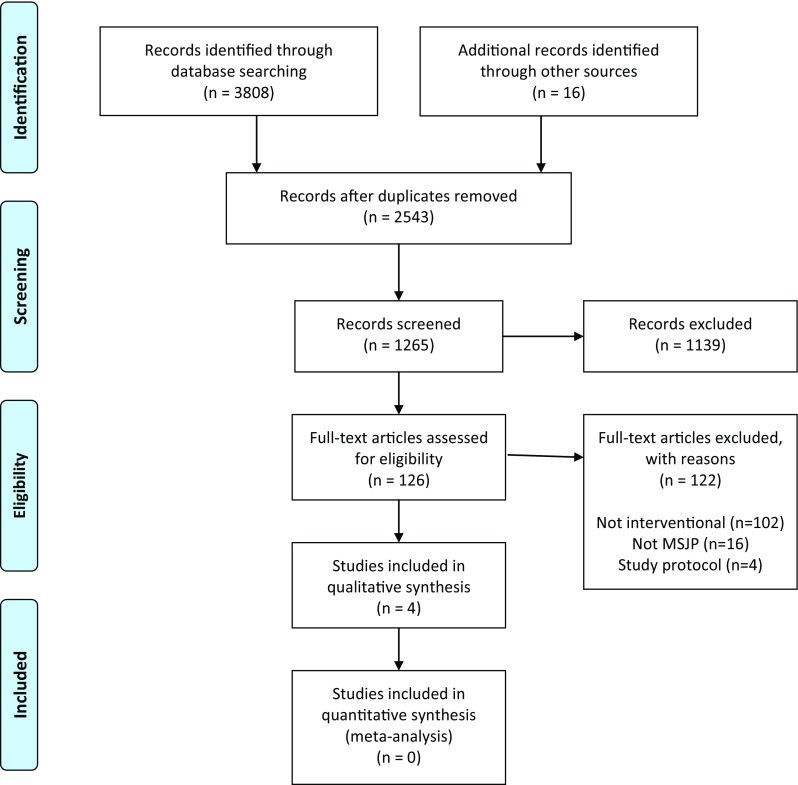
PRSIMA flow-chart of search results

A summary of the search results is presented as Fig. 1. A total of 3824 citations were identified of which 126 were deemed potentially eligible and reviewed at full-text level. The reasons for exclusion included papers not reporting interventional studies (*n* = 102) or they did not include patients who met the a priori definition of MJP (*n* = 16), or were study protocols (*n* = 4). Of the full-text papers reviewed, four satisfied the eligibility criteria and were included.

### Characteristics of included studies

The characteristics of the included studies are presented in Table 2. Three papers reported the findings from observational pre-test/post-test investigations [6, 10, 30], whilst one paper reported a randomised controlled trial (RCT) [31]. Three studies presented data on multidisciplinary team (MDT) programmes for people with MJP [6, 10, 31]. Erol et al. [30] reported the clinical findings of a spa therapy intervention.

**Table 2:** Characteristics of included studies

	Country	*N*	Mean age (range)	Presentation	Intervention	Outcome measures
Erol [30]	France	99	69 (SD: 7)	Generalised OA (two or more joint sites) Diagnosed according to Kellgren–Moore criteria; and/or ACR criteria; and/or Dougados criteria; and/or OA in at least 3 localizations (one can be spine) or OA in 2 localizations with a family history of OA and/or symmetrical articular involvement	18 days Spa therapy (6 days/week) for 3 weeks. Combinations of 6 different treatment modalities (3 for each consecutive day). Treatment modalities were: Berthollet’s technique, Peloidoherapy, Hydrotherapy, Underwater massage, standard massage, water exercise, hydromassage pool, bath with hydro-jets, free immersion in mineral water pool	Patient perception of change in health status; PASS; MHAQ; RAPID 3; VAS Pain; VAS Global Assessment; WOMAC; ODI; EQ-5D; EQ-VAS; OMERACT-OARSI responder criteria set; patient perceived wellbeing; change in drug consumption
Cuperus [31]	Netherlands	147	Gp 1: 61 (SD: 8) Gp 2: 59 (SD:8)	Pain in 2 or more joints. Clinical diagnosis with GOA (defined as: objective signs indicating OA in ≥ 2joint areas; complaints in ≥ 3 joint areas, AND being limited in daily activities – HAQ-DI score ≥ 5)	6-week non-pharmacological MDT programme delivered (1) face-to-face (6–8 group sessions 2–4 Hrs each) vs (2) telephone-based (2 face-to-face sessions PLUS 4 15–30 min telephone sessions) from specialist nurse. Motivational interviewing for improved symptom self-management with support on exercise, physical activity, goal-setting	HAQ-DI; SF-36; General Self-Efficacy Scale; Illness Cognitions Questionnaire; Tampa scale for Kinesiophobia; SQUASH; EQ-VAS; Subjective fatigue subscale of Checklist Individual Strength; Patient specific complaints questionnaire; daily function Likert scale
Lillefjell [6]	Norway	143	45.7 (SD: 8.9)	Pain for a minimum 3 months. 94% MJP	5 week (6 h/day) for 4 week followed by 52 weeks of 6 h/day 1–3 days/week MDT rehabilitation programme (biological, social and psychological)	VAS for pain, physical, cognitive and psychological function and coping. COOP/WONCA chart, HADS, presenteeism
Moradi [10]	Germany	389	44.3 (22–64)	Pain for minimum 3 months in single or Multiple joints	MDT delivered for 3 weeks (biological, Social and psycho-logical)	(MJP presented separately) Pain intensity VAS, SF-36, Funktionsfragebogen Hannover–Rucken (FFbH-R)

*ACR* American College of Rheumatology; *COOP*/*WONCA* Dartmouth Primary Care Cooperative Information Project-World Organization of National Colleges, Academies and Academic Associations of General Practitioners/Family Physicians; *Gp* group; *HADS* hospital anxiety and depression scale; *HAQ-DI* health assessment questionnaire-disability index; *MDT* multi-disciplinary team; *MHAQ* modified health assessment questionnaire; *MJP* multi-joint pain; *OA* osteoarthritis; *ODI* Oswestry Disability Index; *RAPID 3* Routine Assessment of Patient Index Data-3; *PASS* Pain Anxiety Symptom Scale; *SD* standard deviation; *SF-36* Short Form-36; *SQUASH* Short Questionnaire to Assess Health-enhancing physical activity; *VAS* visual analogue scale

Two studies characterised and termed their cohorts as people with ‘generalised osteoarthritis’ [30, 31]. Lillefjell et al. [6] and Moradi et al. [10] termed this group ‘chronic musculoskeletal pain’ but provided specific definitions of joint involvement, thereby meeting the eligibility of MJP for this review. In all four studies, the definition of joint pain in two or more joints were specified (Table 2). Moradi et al. [10] and Lillefjell et al. [6] recruited people with both single and MJP. However, Moradi et al. [10] reported the findings of single- and MJP separately, whilst 94% of Lillefjell et al’s [6] cohort were MJP and, therefore, met the eligibility criteria.

### Quality assessment

A summary of the critical appraisal results is presented in Table 3. The randomised controlled trials presented with a moderate risk of bias, whilst the non-randomised controlled trials presented with ‘low’ quality evidence. In both, the TIDieR checklist assessment (Table 4) highlighted recurrent limitations in intervention reporting. The included studies were consistently poor in reporting materials and participant’s role within and towards the intervention and its delivery (Item 3; 25%), the description of the intervention’s activities (Item 4; 25%), the dosage (Item 8; 0%), the adaption or modification of the intervention (Item 9 and 10; 0%) and only Cuperuset al [31] reported the fidelity (Item 12; 25%). Based on the quality assessment, seven key research design considerations where identified for consideration when designing future trials for people with MJP. These are presented in Table 5.

**Table 3:** Results of the quality assessment evaluation

	Downs and black critical appraisal checklist items																										
	1	2	3	4	5	6	7	8	9	10	11	12	13	14	15	16	17	18	19	20	21	22	23	24	25	26	27
Cuperus [31]	1	1	1	1	1	1	1	1	1	0	1	1	1	0	1	NA	0	1	0	1	1	1	1	1	1	1	0
Erol [30]	1	1	1	1	1	1	0	1	0	1	1	1	1	0	0	NA	0	1	1	1	NA	NA	0	0	0	1	0
Lillefjell [6]	1	1	1	0	1	1	1	0	1	1	1	1	1	0	0	NA	1	1	0	1	NA	NA	0	NA	0	1	0
Moradi [10]	1	1	1	1	2	1	1	0	0	1	1	1	1	0	0	NA	0	1	0	1	NA	NA	0	NA	0	0	0
Total per item (%)	100	100	75	75	50	100	100	50	25	100	100	100	75	0	25	100	25	100	25	100	NA	NA	25	NS	25	50	0

Items: Is the hypothesis/aim/objective of the study clearly described? Are the main outcomes to be measured clearly described in the “Introduction” or “Methods” section? Are the characteristics of the patients included in the study clearly described? Are the interventions of interest clearly described? Are the distributions of principal confounders in each group of subjects to be compared clearly described? Are the main findings of the study clearly described? Does the study provide estimates of the random variability in the data for the main outcomes? Have all important adverse events that may be a consequence of the intervention been reported? Have the characteristic of patients lost to follow-up been described? Have actual probability values been reported (e.g. 0,035 rather than < 0.05) for the main outcomes except where the probability value is less than 0.001? Were the subjects asked to participate in the study representative of the entire population from which they were recruited? Were those subjects who were prepared to participate representative of the entire population from which they were recruited? Were the staff, places and facilitates where the patients were treated, representative of the treatment the majority of patients received? Was an attempt made to blind study subjects to the intervention they have received? Was an attempt made to blind those measuring the main outcomes of the intervention? If any of the results of the study were based on “data dreading” was this made clear? In trials and cohort studies, were the analyses adjust for different lengths of follow-up of patients, or in case-control studies, is the time period between the intervention and outcome the same for cases and controls? Were the statistical tests used to assess the main outcome appropriate? Was compliance with the intervention/s reliable? Were the main outcome measures used accurate (valid and reliable)? Were the patients in different intervention groups (trials and cohort studies) or were the cases and controls (case–control studies) recruited from the same population? Were study subjects in different intervention groups (trials and cohort studies) or were the cases and controls (case–control studies) recruited over the same time? Were study subjects randomized to intervention groups? Was the randomized intervention assignment concealed from both patients and health care staff until recruitment was complete and irrevocable? Was there adequate adjustment for confounding in the analyses from which the main findings were drawn? Were losses of patients to follow-up taken into account? Did the study have sufficient power to detect a clinically important effect where the probability value for a difference being due to chance < 5%

1—satisfied, 2—completely satisfied when score is out of 2 for item 5, 0—not satisfied, NA—not applicable due to study design

**Table 4:** TIDieR checklist for interventional reporting

	TIDieR checklist items												Total per study (%)
	1	2	3	4	5	6	7	8	9	10	11	12	
Cuperus [31]	1	1	0	0	1	1	0	0	0	0	1	1	50
Erol [30]	1	1	1	1	1	0	1	0	0	0	1	0	58.3
Lillefjell [6]	1	1	0	0	0	0	0	0	0	0	0	0	16.7
Moradi [10]	1	1	0	0	0	1	0	0	0	0	0	0	25
Total per item (%)	100	100	25	25	50	50	25	0	0	0	50	25	

Checklist items: Provide the name or a phrase that describes the intervention. Describe any rationale, theory, or goal of the elements essential to the intervention. Describe any physical or informational materials used in the intervention, including those provided to participants or used in intervention delivery or in training of intervention providers. Provide information on where the materials can be accessed (e.g. online appendix, URL). Describe each of the procedures, activities, and/or processes used in the intervention, including any enabling or support activities. For each category of intervention provider (e.g. psychologist, nursing assistant), describe their expertise, background and any specific training given. Describe the modes of delivery (e.g. face-to-face or by some other mechanism, such as internet or telephone) of the intervention and whether it was provided individually or in a group. Describe the type(s) of location(s) where the intervention occurred, including any necessary infrastructure or relevant features. Describe the number of times the intervention was delivered and over what period of time including the number of sessions, their schedule, and their duration, intensity or dose. If the intervention was planned to be personalised, titrated or adapted, then describe what, why, when, and how. If the intervention was modified during the course of the study, describe the changes (what, why, when, and how). Planned: If intervention adherence or fidelity was assessed, describe how and by whom, and if any strategies were used to maintain or improve fidelity, describe them. Actual: If intervention adherence or fidelity was assessed, describe the extent to which the intervention was delivered as planned

## Intervention 1: Multi-disciplinary programme

Three studies presented data on MDT programmes in people with MJP [6, 10, 31].

### Pain

Two studies of moderate [31] and weak [6] evidence were available to support a significant decrease in pain at short-term follow-up. Cuperus et al. [31] reported a significant decrease in pain by 3.2 points at 6-week follow-up. Lillefjell et al’s [6] MDT programme reported a mean decrease in pain by 3.8 points (*p* < 0.05) at 5-week follow-up. Moderate evidence was available from Cuperus et al. [31] of no statistically significant change in pain score for their telephone-based intervention (6 week difference: 0.96; 1-year: 0.76).

There was weak evidence from one study [10] to support a significant decrease in pain at mid-term follow-up (6 months). Moradi et al. [10] reported a mean decrease of 1.4 points (*p* < 0.001) for their dual-joint pain group, and 1.2 points for their MJP group (*p* < 0.001).

Two studies of moderate [31] and weak [6] evidence were available to support a significant decrease in pain at long-term follow-up. Cuperus et al. [31] reported a 2.8 point decrease at 1-year (*p* < 0.05) for their face-to-face MDT intervention and Lillefjell et al. [6] 5.7 points (*p* < 0.01) at long-term (57 week) follow-up.

### Physical function

Two studies of moderate [31] and weak [6] quality were available to evaluate physical function. Cuperus et al. [31] reported a statistically significant increase in physical function for their cohort who received the face-to-face and telephone-based MDT interventions at 6 weeks (mean difference form baseline: 2.25 and 1.58, respectively; *p* < 0.05). Similarly, Lillefjell et al. [6] reported a mean difference in COOP/WONCA daily activity assessment but only by 0.10 points and 0.23 points at 5 and 57 weeks follow-up (*p* < 0.001).

There was weak evidence from one study [10] to support improvements at mid-term follow-up. Moradi et al. [10] reported a mean increase in SF-36 physical function of 17.1 points specifically in their dual-joint pain cohort and 9.2 points in their MJP cohort at 6-month follow-up (*p* < 0.001).

There was conflicting evidence from one study of moderate [31] and another of weak quality [6] regarding functional outcomes in the longer-term. Cuperus et al. [31] reported neither intervention provided statistically significant findings from baseline for this measure at 12 months (SF-36 physical function: mean difference from baseline: 1.58 points and 1.13 points; *p* > 0.05). Lillefjell et al. [6] reported statistically significant improvements in physical function at longer-term follow-up assessments.

### Health-related quality of life

One study [31] of moderate evidence supported improvements in quality of life using the EQ-VAS. Cuperus et al. [31] reported a significant improvement in quality of life in both intervention groups at 6-week follow-up (*p* < 0.05), the magnitude of improvement being greater for the face-to-face group (mean difference: 8.43 points) compared to the telephone-based intervention group (mean difference 5.22 points). At 12 months, this difference remained in the face-to-face group (mean difference 6.59; *p* < 0.05), but not in the telephone-based intervention group (mean difference 3.73).

### Anxiety and depression

One study [6] of weak evidence assessed anxiety and depression at short and long-term follow-up using the HADS. Lillejjfell et al. [6] reported that both anxiety (*p* < 0.05) and depression (*p* < 0.01) both significantly decreased at the 5 and 57-week follow-up intervals in this MDT intervention. This was not a large change. There was a mean reduction in anxiety by 0.18 points at 5 weeks, and 0.9 at 57 weeks. Similarly, there was a mean reduction in depression by 0.44 points at 5 weeks, and 0.95 at 57 weeks.

### Fear avoidance and kinesophophia

There was moderate evidence from one study [31] showing there was no statistically significant difference in kinesophobia in either the face-to-face MDT intervention or telephone-based intervention at 6 weeks or 12 months (*p* > 0.05).

### Self-efficacy

There was moderate evidence from one study [31] reporting no statistically significant difference in self-efficacy when evaluated using the General Self-Efficacy Questionnaire in either the face-to-face MDT intervention or telephone-based intervention at 6 weeks or 12 months (*p* > 0.05).

### Missing outcomes

No studies reported findings of fear avoidance, absenteeism or presenteeism for either of the three MDT intervention papers.

## Intervention 2: Spa therapy

One study presented outcomes of a spa therapy intervention for people with MJP [30]. Using a Best Evidence Synthesis approach, the outcomes from this study were classified as ‘weak’ evidence.

### Pain

Whilst there was a statistically significant improvement in pain from baseline to post-treatment (4.8 to 3.7; *p* < 0.01), this was not statistically significant at mid-term (8 months) follow-up (4.8 to 4.5; *p* = 0.15).

### Physical function

There was a statistically significant improvement in physical function when measured using the WOMAC subsection from baseline to post-treatment (19.5–15.2; *p* < 0.01). This was not statistically significant at mid-term (8 months) follow-up (19.5–19.7; *p* = 0.76). The Oswestry Disability Index decreased in the immediate-term (3 weeks) from 25.4 to 20.8 (*p* < 0.01) and at the mid-term but only differed by two points (*p* = 0.04).

### Health-related quality of life

Immediate-term (3-week commencement of treatment), 33% reported an acceptable symptom state (achieved PASS). Mid-term (8-month post-commencement of treatment) this increased to 75%. There was a significant increase in the Modified Health Assessment Questionnaire (*p* = 0.03), Routine Assessment of Patient Index Data (*p* < 0.01), EQ-5D (0 = 0.02), EQ VAS (*p* < 0.01) and Patient Global Assessment (*p* < 0.01). However, none of these were statistically significant at the mid-term (final) assessment (*p* > 0.05).

### Other measures

No data was reported on measures including anxiety and depression, fear avoidance, kinesphophia, self-efficacy, absenteeism or presenteeism measured.

## Discussion

Face-to-face MDT rehabilitation interventions may reduce pain, increase function and improve symptom control for people with MJP, and spa-based treatments may result in short-term reductions in symptoms but have limited longer-term benefits. However, the data were of insufficient quality to provide conclusive evidence of effectiveness because of underpowered cohorts, and limitations in reporting of the diagnostic criteria of MJP, intervention procedures or outcome measures used. It is, therefore, not possible to form recommendations on what interventions or packages of care should be used for people with MJP.

Lack of detailed reporting of interventions is a common limitation in rehabilitation trials [32]. Consequently, interventions which have demonstrated effectiveness cannot be readily adopted into clinical practice, and researchers are unable to replicate, develop and synthase research findings. Only one study in this review involving an MDT programme [31] satisfied the criteria for completeness of intervention reporting according to the TIDieR checklist [15]. Detailed reporting is particularly important for the trials of MJP where the interventions proposed are frequently packages of care which are multi-componented, and offer a range of potential interventions, dosage and settings for delivery. Accordingly, researchers and journal reviewers/editors should be mindful of this when reporting and preparing papers for publication to ensure future trials better describe these interventions.

Conducting this review was complicated by the lack of standardised diagnostic criteria and terminology for people with MJP. This may be partly attributed to the nature of MJP as it represents a complex mix of osteoarthritis, back pain and soft tissue disorders [5]. The most commonly adopted term in the current literature is ‘generalised osteoarthritis’ [30, 31], but other terms include ‘widespread musculoskeletal joint pain’ [3, 6, 10], ‘multi-joint site pain’ [9] and ‘multi-site joint pain’ [5, 33]. Furthermore, a range of different radiological and clinical diagnostic criteria have been used to define MJP [30]. However, there is still no consensus on how to define or classify MJP [34, 35]. Adoption of an agreed term to define this population and use of standardised diagnostic criteria for MJP for future trials will be imperative to improve reporting and implementation.

Outcome assessment of people with MJP can be particularly challenging because of disparate clinical symptoms including pain, fatigue, atrophy and psychosocial traits, and also the varied number and location of joints affected by pain [5] and functional restrictions [1]. This review highlights the current lack of standardisation in outcome measures used in MJP trials. Given the variability in population characteristics and presentation, routine use of outcome measures which examine the global impact of MJP should be considered, such as the HAQ Disability Index (HAQ-DI) validated by Cuperus et al. [1]. Moreover, given that MJP can affect people across the adult life-span, routine inclusion of outcome tools which assess work-related productivity (presenteeism as well as absenteeism) such as the Work Productivity and Activity Impairment Questionnaire [36] would also be valuable for future trials.

This study has two limitations of note. First, due to the limited evidence, it was not possible to answer the original research question on the effectiveness of interventions for MJP. Second, due to the variability in diagnostic criteria used and poor study reporting, the review team found the identification of eligible papers a challenge. Strategies including regular discussions on eligibility, member-checking and consensus adjudicators were used to overcome this. Nonetheless, until there is an agreed diagnostic terminology used to classify this population, future systematic reviewers may face similar difficulties. Given the limitations in reporting the diagnostic criteria for MJP and the interventions which have been investigated, we have developed research design considerations (Table 5) to aid the future development of trials which are urgently required to better treat this clinical population.

**Table 5:** Research design considerations for the design of trials on MJP

Standardised definition and criteria for determining the presence of MJP where participants present with two or more joint ‘regions’ concurrently painful
Trials should record and document the location and frequency of joint sites involved for each trial participant
Interventions aim to treat all painful joint sites participants present with rather than individual joints
Dose, frequency and any modification or tailoring of treatments should be considered to allow flexibility in intervention prescription given the heterogeneity in the MJP population’s symptoms. Decision-making for adaptation of interventions should be presented
All co-interventions should be reported for both experimental or control intervention trial-arms and decision-making for when co-interventions are prescribed should be considered
Outcome measures should be selected to evaluate global health status (symptoms and function) rather than site-specific outcomes
Outcome reporting should be catagorised accordingly to the location and number of joint pain sites participants present with

In conclusion, there is insufficient literature to make clinical recommendations on the treatment of people with MJP. The current evidence-base is limited by study design, diagnostic classification, selection of standardized outcome measures and reporting of study interventions. Given the significant health burden which MJP has on both the individual and wider society, developing and testing interventions to improve symptom management of this condition is a research priority. The methodological considerations highlighted on design and reporting should be considered when developing such trials.
